# Medical Aid in Dying: A Societal Challenge

**DOI:** 10.3389/ijph.2024.1608151

**Published:** 2025-01-07

**Authors:** Uwe Güth, Iris D. Hartog, Andres R. Schneeberger

**Affiliations:** ^1^ Faculty of Medicine, University of Basel, Basel, Switzerland; ^2^ Brust-Zentrum Zürich, Zurich, Switzerland; ^3^ Center of Expertise in Palliative Care, Leiden University Medical Center (LUMC), Leiden, Netherlands; ^4^ Department of Psychiatry, University of California San Diego, La Jolla, CA, United States

**Keywords:** medical aid in dying, assisted dying, assisted suicide, end-of-life decisions, palliative care

Medical aid in dying (MAID) has emerged as one of the most complex and emotionally charged topics in healthcare [1–4]. While proponents argue that it offers a compassionate option for those facing unbearable suffering, critics raise concerns about the possibility for abuse and how this might impact society’s most vulnerable members. To truly understand the implications of MAID, we must not only follow the journey of that the person requesting MAID but also that of the others involved: patients, families, healthcare providers, and society at large. In this special issue, several themes and perspectives regarding MAID are highlighted from different countries (see Table 1).

**TABLE 1:** Medical aid in dying around the world (adapted from [5]).

Only a few countries or federated states across the world have laws that allow various forms of MAID [6-9]^1^. **AS:** Assisted suicide; doctors are permitted to prescribe lethal drugs for self-administration. The critical point is that patients, voluntarily wishing to die, must carry out the final act of the procedure themselves. **VAE:** Voluntary active euthanasia; in addition to AS, doctors or healthcare professionals are permitted to also administer the lethal drug.	
	**Country/federal stateLegal form: AS or VAE, year of legalization**
Terminal Illness Requirement (TIR) MAID is reserved to patients suffering from a terminal illness and are expected to die naturally in the immediate future. This is usually defined as death occurring in no more than 6 months	USA: AS. Oregon (1997), Washington state (2008), Montana (2009) Vermont (2013), California (2016), Colorado (2016), Maine (2019) Hawaii (2019), New Jersey (2019), New Mexico (2021); Washington D.C. (2016) New Zealand: VAE, 2021 Australia: VAE.^2^ Victoria (2019), Western Australia (2021), Tasmania (2022), Queensland (2023), South Australia (2023), New South Wales (2023), Australian Capital Territory (will go into effect in 11/2025)
No Terminal Illness Requirement Not only illnesses leading to death in the foreseeable future, but also symptom-oriented conditions are accepted as criteria for granting MAID, e.g., symptoms of illness and/or functional limitations are present that are the cause of intolerable suffering with no prospect of improvement	Switzerland: AS, 1942; first case of MAID occurred in 1985 TIR was removed from medical-ethical guidelines in 2018^3^ Columbia: VAE, 1997; TIR was removed from regulations in 2021 The Netherlands: VAE, 2001 Belgium: VAE, 2002 Luxembourg: VAE, 2009 Spain: VAE, 2021 Canada: VAE, 2016; TIR was removed from regulations in 2021 Austria: AS, 2022 Portugal: AS, 2023^4^

1 In some Western countries such as Germany, France, Italy and Great Britain, there are active discussions about implementing MAID. however, their parliaments or governments have not legislated or regulated the practice yet.

2 Australia: As of June 2024 all states and the Australian Capital Territory have passed legislation. MAID is not legalized in the Northern Territory.

3 Switzerland: There is no specific legislation regulating how MAID should be practiced.

4 Portugal: VAE is only allowed in cases where AS is impossible due to a physical incapacitation of the patient.

Starting at the center of MAID’s focus, there are the individuals who seek this option. Research by Currin-McCulloch et al. (United States) provide valuable insights into the decision-making processes of these patients. Their study reveals that the motivation for a patient to request MAID is far from simple or straightforward. It is a highly personal decision-making process that rests against personal experiences that are greatly impacted by societal controversy and social pressure in the United States. This context, potentially adding to the tensions intrinsic to the process of seeking MAID, underscores the need for robust support and care systems with clear communication between patients, families, and healthcare professionals.

The support needs of patients and their relatives throughout MAID trajectories is addressed by Vissers et al. (Belgium). Their qualitative study identified eight categories of assistance needs, from facilitating social interaction and handling organizational and practical issues to support for relatives to understand the patient’s desire for MAID. As the authors conclude that patients and relatives experience the MAID trajectory as social and existential rather than only medical, patients requesting MAID and their relatives may benefit from a palliative care approach, just like patients and their relatives in other end-of-life trajectories. An integrated approach of MAID and palliative care seems warranted, rather than viewing them as conflicting paths for end-of-life care.

The importance of family dynamics and support networks in MAID trajectories is also reflected in a study by Sperling (Israel) on family members and close friends that support patients who travel to Switzerland for MAID. This study highlights the complex emotional landscape navigated by loved ones during this process. Families often struggle with supporting and facilitating the patient’s request, the impact of traveling to another country with entirely different surroundings, and handling procedures after the patient’s death. These findings show several unfulfilled support needs of families and other loved ones that are also present in the study by Vissers et al., but in the case of “suicide tourism” these inadequacies are even more obvious.

As some of the countries with MAID legislation do not require patients to have a lethal disease to be eligible for MAID, special considerations arise regarding MAID requests from patients with age-related health problems. Kraak-Steenken et al. (The Netherlands) found that people with an accumulation of health problems related to old age that requested assisted dying most often had osteoarthritis, vision and/or hearing impairment. Their primary reasons for requesting assisted dying included physical decline, dependency, general weakness/fatigue and (fear of) losing control of one’s life. The researchers also found that patients with certain characteristics, e.g., care dependence, disability/immobility, loss of control and a treatment relationship with the physician longer than a year, were more likely to have their request granted. In contrast, having “no purpose in life” or “not wanting to be a burden” lowered the likelihood of a granted request for MAID. These findings raise challenging questions about the nature of suffering, the role of family and society and how physicians deal with individuals handle those wishing to die due to an accumulation of age-related conditions. Greater understanding of these topics can contribute to the ongoing debate on the acceptability of MAID for people without life-threatening conditions.

MAID requests in countries with MAID legislation also place significant demands on healthcare professionals navigating the patient’s journey. Research by Perron et al. (Canada) underscores the legal, administrative, clinical, emotional and ethical challenges faced by physicians and other healthcare professionals involved in MAID, especially in countries with increasing counts of MAID requests. They examined interdisciplinary support groups for professionals involved in MAID in Quebec, and found that they vary significantly. The working practices of the organizations ranged from acting as a central point of contact that is entirely responsible throughout end-of-life procedures, to providing support only. A “middle ground” between these two positions was preferred, in which the MAID trajectory is the doctor’s responsibility but support is provided by a team. Although support groups like these require adaptation to the specific reality of every context, this study provides valuable insights for the development of support structures for healthcare providers involved in MAID.

One often overlooked aspect of the MAID journey is the need for comprehensive aftercare for bereaved relatives and healthcare workers. Renckens et al. (Netherlands) shed light on current practices in such provisions following euthanasia or physician-assisted suicide. Aftercare focused on practical aspects of the MAID journey, the emotional experience of relatives during the MAID trajectory, and relatives' current mental wellbeing. Their findings also reveal significant gaps in support for these families post-MAID. The authors conclude that aftercare conversations with a physician covering a wide-range of topics are likely to be valuable for all bereaved relatives, and not just for “at risk” populations typically targeted by policies and guidelines.

In conclusion, in the light of the different MAID legislation, policies and practices around the world, the presented studies provide a small snapshot of a very complex and important aspect of healthcare. As we look to the future of MAID, it is clear that a holistic and interdisciplinary approach is needed to support not only the person seeking to enter this journey but also family, friends and healthcare professionals. This complex issue touches on medicine, ethics, law, psychology, and sociology, among other disciplines. Future research should focus on expanding our understanding of the diverse practices, contexts, and implications related to end-of-life care for those wishing to die, families, the bereaved, physicians, and other healthcare professionals. It is crucial to gain deeper insights into current practices and legislation, allowing for critical analysis and identification of areas for improvement. This research should adopt an international perspective and consider cultural aspects to develop a more comprehensive understanding of the field.
